# Chick chorioallantoic membrane assay as an *in vivo* model to study the effect of nanoparticle-based anticancer drugs in ovarian cancer

**DOI:** 10.1038/s41598-018-25573-8

**Published:** 2018-06-04

**Authors:** Binh Thanh Vu, Sophia Allaf Shahin, Jonas Croissant, Yevhen Fatieiev, Kotaro Matsumoto, Tan Le-Hoang Doan, Tammy Yik, Shirleen Simargi, Altagracia Conteras, Laura Ratliff, Chiara Mauriello Jimenez, Laurence Raehm, Niveen Khashab, Jean-Olivier Durand, Carlotta Glackin, Fuyuhiko Tamanoi

**Affiliations:** 1 Department of Microbiology, Immunology and Molecular Genetics, Jonsson Comprehensive Cancer Center, University of California, Los Angeles, CA USA; 2 Department of Developmental and Stem Cell Biology, City of Hope-Beckman Research Institute, Duarte, CA USA; 3 Smart Hybrid Materials Laboratory (SHMs), King Abdullah University of Science and Technology, Thuwal, Saudi Arabia; 4 Institute for Integrated Cell-Material Sciences, Institute for Advanced Study, Kyoto University, Kyoto, Japan; 5 Institut Charles Gerhardt Montpellier, UMR-5253 CNRS-UM2-ENSCM-UM1, Montpellier, France; 6 Present Address: Laboratory for Stem Cell Research and Application, Vietnam National University-Ho Chi Minh City, Ho Chi Minh City, Vietnam; 7 Present Address: Center for Micro-Engineered Materials, Advanced Materials Laboratory, University of New Mexico, Albuquerque, New Mexico USA; 8 Center for Innovative Materials and Architectures, Vietnam National University-Ho Chi Minh City, Ho Chi Minh City, Vietnam

## Abstract

New therapy development is critically needed for ovarian cancer. We used the chicken egg CAM assay to evaluate efficacy of anticancer drug delivery using recently developed biodegradable PMO (periodic mesoporous organosilica) nanoparticles. Human ovarian cancer cells were transplanted onto the CAM membrane of fertilized eggs, resulting in rapid tumor formation. The tumor closely resembles cancer patient tumor and contains extracellular matrix as well as stromal cells and extensive vasculature. PMO nanoparticles loaded with doxorubicin were injected intravenously into the chicken egg resulting in elimination of the tumor. No significant damage to various organs in the chicken embryo occurred. In contrast, injection of free doxorubicin caused widespread organ damage, even when less amount was administered. The lack of toxic effect of nanoparticle loaded doxorubicin was associated with specific delivery of doxorubicin to the tumor. Furthermore, we observed excellent tumor accumulation of the nanoparticles. Lastly, a tumor could be established in the egg using tumor samples from ovarian cancer patients and that our nanoparticles were effective in eliminating the tumor. These results point to the remarkable efficacy of our nanoparticle based drug delivery system and suggests the value of the chicken egg tumor model for testing novel therapies for ovarian cancer.

## Introduction

Annually, ovarian cancer accounts for an estimated 239,000 new cases and 152,000 deaths worldwide annually^1,2^. This disease has both the highest morbidity and mortality rate among cancers of the reproductive system. Each year, at least 20,000 women in the United States are diagnosed with ovarian cancer^3^. Recurrent epithelial ovarian cancer (EOC) is almost uniformly lethal. Tumor recurrence and metastasis are primarily responsible for the 70% five-year mortality of advanced EOC. Despite successful initial surgery and chemotherapy, over 70% of advanced EOC will recur, and only 15–30% of recurrent disease will respond to chemotherapy^3,4^. Moreover, drug resistance causes treatment failure in over 90% of patients with the metastatic disease^5^. Consequently, recurrent metastatic disease is a major clinical challenge that lacks effective therapy. Thus, there is a clear need to develop new therapies for this cancer.

To initiate development of a novel therapy, it is necessary to employ a tumor model to evaluate its efficacy. In this paper, we discuss the chicken egg tumor model that possesses advantageous features as a tumor model. The chicken chorioallantoic membrane (CAM) system has been widely used for the study of human tumor growth^6–12^. In this model, human cancer cells are transplanted onto the CAM membrane that surrounds chicken embryo within the fertilized chicken egg. After three days, a tumor that contains human tumor features including highly vascularized structure, presence of multiple types of cells and extracellular matrix, is formed. Formation of tumor in the chicken eggs that possesses features that resemble tumor microenvironment contrasts with cancer cell derived models such as tumor organoid^13,14^. The rapid tumor formation contrasts with the long period needed to form tumors in mice. The formation of a tumor reflects incomplete development of the immune system at this stage of chicken development^6,15,16^ and the rich nutrient conditions of the CAM membrane enhanced by its rich angiogeneic features. Fertilized chicken eggs are also much less expensive than immune-compromised mice. Furthermore, less animal oversight is needed for this system, since eggs are not considered “animal” yet. These characteristics point to attractive features of this animal model system.

Mesoporous silica nanoparticles (MSNs) have emerged as a powerful drug delivery vehicle. These silica particles have a diameter ranging from 50 to 400 nm and contain thousands of pores where anticancer drugs can be stored. Various cellular and animal studies have been reported that point to the efficacy of MSNs to deliver anticancer drugs^17–28^ MSNs also provide an effective vehicle for delivery of nucleic acid agents such as siRNA in cells and animal models^29–31^. Because of the relative stability of this nanomaterial, various chemical modifications can be applied to these nanoparticles. A surface charge property can be altered to prevent the aggregation of particles in biological media and to achieve prolonged circulation. Furthermore, various nanomachines can be engineered on the particle surface to confer the ability to release anticancer drug upon stimuli including light and magnetic field exposure^31–36^.

More recently, it has been shown that MSNs can be tuned for biodegradation by incorporating biodegradable bonds within the framework of the nanoparticle^37–41^. We and others have recently described a new generation of mesoporous nanoparticles called biodegradable PMO (Periodic Mesoporous Organosilica) nanoparticles which are tuned for biodegradation by incorporating disulfide, tetrasulfide bonds or protease sensitive bonds leading to excellent candidates for nanomedicine applications^37,40^. These nanoparticles retain the advantageous features of MSNs described above and, in addition, they possess enhanced degradability. The biodegradable PMOs exhibit efficient loading capacity with anticancer drugs such as doxorubicin and the cargo can be delivered into human cancer cells. However, no extensive animal experiments have been carried out with these new nanoparticles.

In this paper, we establish the chicken egg tumor model using ovarian cancer cells. We demonstrate that the tumors formed closely resemble human tumor pathology. Histopathological analyses show that the tumors contain extracellular matrix and stromal cells. Intravenous injection of biodegradable PMO nanoparticles loaded with doxorubicin results in tumor elimination without major adverse effects, which contrasts with the use of free doxorubicin. We further confirm that doxorubicin is delivered specifically to tumor. Tumor accumulation of the nanoparticles is observed. We further show that the same treatment can be carried out with patient-derived xenografts formed by transplanting ovarian cancer patient tumor into the CAM model.

## Results

### Establishing an ovarian cancer model using the chicken egg CAM system

**Figure 1:**
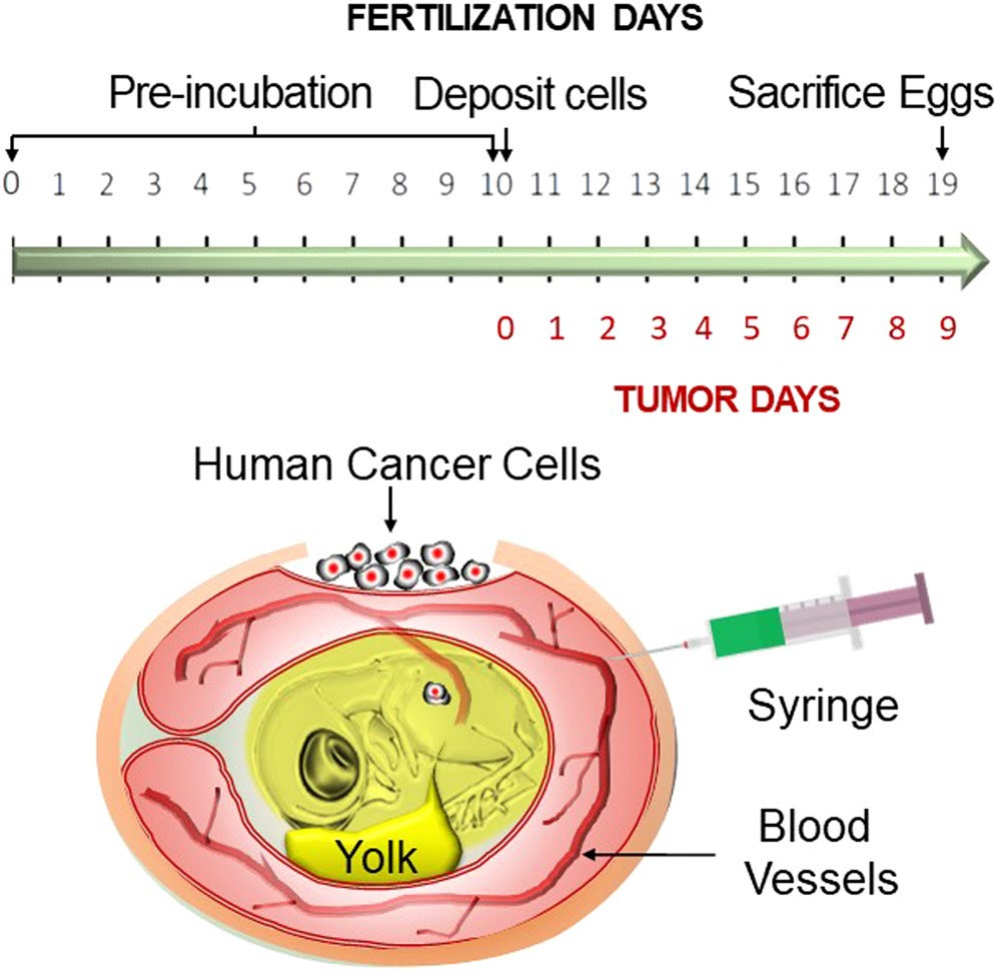
A timeline of the experiment. Fertilized eggs are incubated for ten days at which time human cancer cells are transplanted onto the CAM membrane. Tumor is formed three days later. Nanoparticles loaded with anticancer drug are injected into the vein and effect on tumor growth is examined.

**Figure 2:**
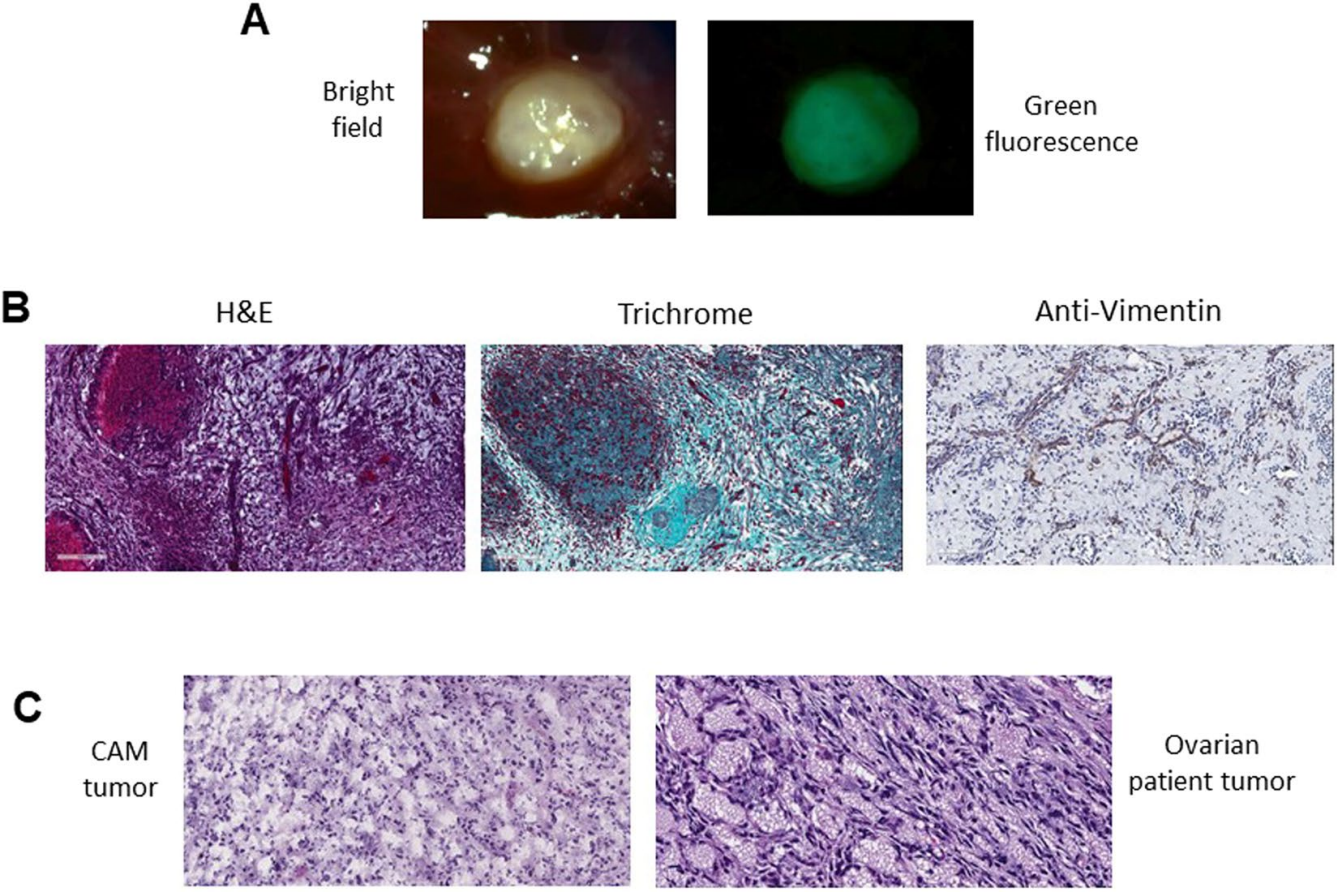
Characterization of ovarian tumor established on the CAM membrane. (**A**) Tumor formed on the CAM membrane by transplanting Ov8GFP cells. (**B**) H&E, trichrome and anti-Vimentin staining of the Ov8GFP tumor. (**C**) Comparison of the CAM tumor and ovarian patient tumor after H&E staining.

To establish an ovarian cancer chicken egg model, we used a human cancer cell line Ov8GFP derived from OVCAR-8. As shown in Fig. 1, fertilized eggs were incubated for ten days. At this time, the embryo is surrounded by the CAM membrane that provides nutrients to the embryo. A window was opened on the egg shell to allow the transplantation of human cancer cells. Approximately two million cells were transplanted onto the CAM membrane using a Teflon ring on the CAM membrane to provide a space to position the cancer cells. By day 13, we observed tumor masses appearing on the CAM membrane. Figure 2A shows the tumor formed on the CAM membrane. Since Ov8GFP cells are engineered to express GFP, the tumor formed can be detected by the green fluorescence of GFP.

Histopathological analysis was carried out to characterize the tumor formed on the CAM membrane. As shown in Fig. 2B, trichrome staining revealed the presence of extracellular matrix. Anti-vimentin staining depicting the presence of stromal cells was revealed by the presence of brown IHC staining. We also compared the tumor formed on the CAM membrane with histological slices of tumor from cancer patient. The Ov8GFP tumor from the CAM model was embedded in paraffin and thin sections were cut and transferred onto slides followed by Pathology analysis. Tumors from an ovarian cancer patient was processed in a similar manner and compared. As shown in Fig. 2C, H&E staining of these samples exhibited striking resemblance including the presence of cells that are densely populated. Thus, the tumor formed on the CAM membrane closely resembles the ovarian cancer patient tumor.

### Biodegradable PMO nanoparticles

**Figure 3:**
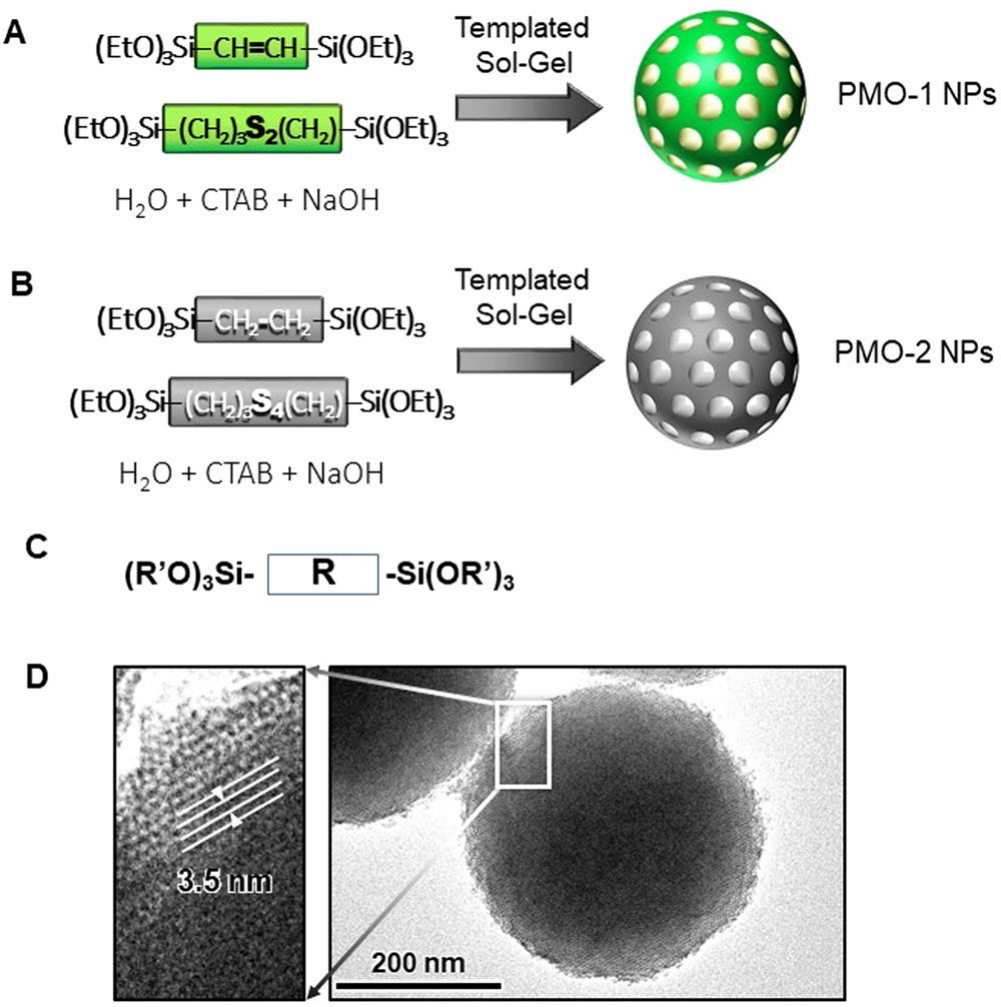
Biodegradable PMO nanoparticles used in the study. PMO-1 contains disulfide bonds (**A**) and PMO-2 contains tetrasulfide bonds (**B**). (**C**) A general structure of precursor molecules used for the synthesis. (**D**) TEM picture of PMO-2 nanoparticle.

We have recently developed a new generation of mesoporous silica nanoparticles called biodegradable PMOs^37,40^. These nanoparticles contain biodegradable bonds within the framework of mesoporous silica network and thus are degradable by conditions inside the cell such as reducing environment. In this work, we used two types of biodegradable PMO nanoparticles containing disulfide (PMO-1) or tetrasulfide bond (PMO-2). PMO-1 and PMO-2 were synthesized using the sol-gel synthesis method with precursors containing disulfide or tetrasulfide bond (Fig. 3A–C). The use of these special precursors (Fig. 3C) for synthesis results in the incorporation of these biodegradable bonds into the framework of nanoparticles. During the synthesis, surfactant CTAB (cetyltrimethylammonium bromide) was included to produce porous structure. PMO-1 nanoparticles were synthesized by mixing bis(triethoxysilylpropyl)disulfide with bis(triethoxysilyl)ethylene (50–50 ratio)^37^. The resulting nanoparticles have a diameter of 200 nm and contain pores with 2 nm diameter^37^. PMO-2 nanoparticles were synthesized by mixing bis(triethoxysilylpropyl)tetrasulfide with bis(triethoxysilyl)ethane (50–50 ratio). The surface of this nanoparticle was modified with phosphonate to increase negative charge of the surface. TEM picture of PMO-2 is shown in Fig. 3D. Pores with a diameter of 3.5 nm is visible. PMO-2 is slightly larger than PMO-1 with a diameter of approximately 330 nm.

### Intravenous injection of biodegradable PMO loaded with doxorubicin results in tumor elimination

**Figure 4:**
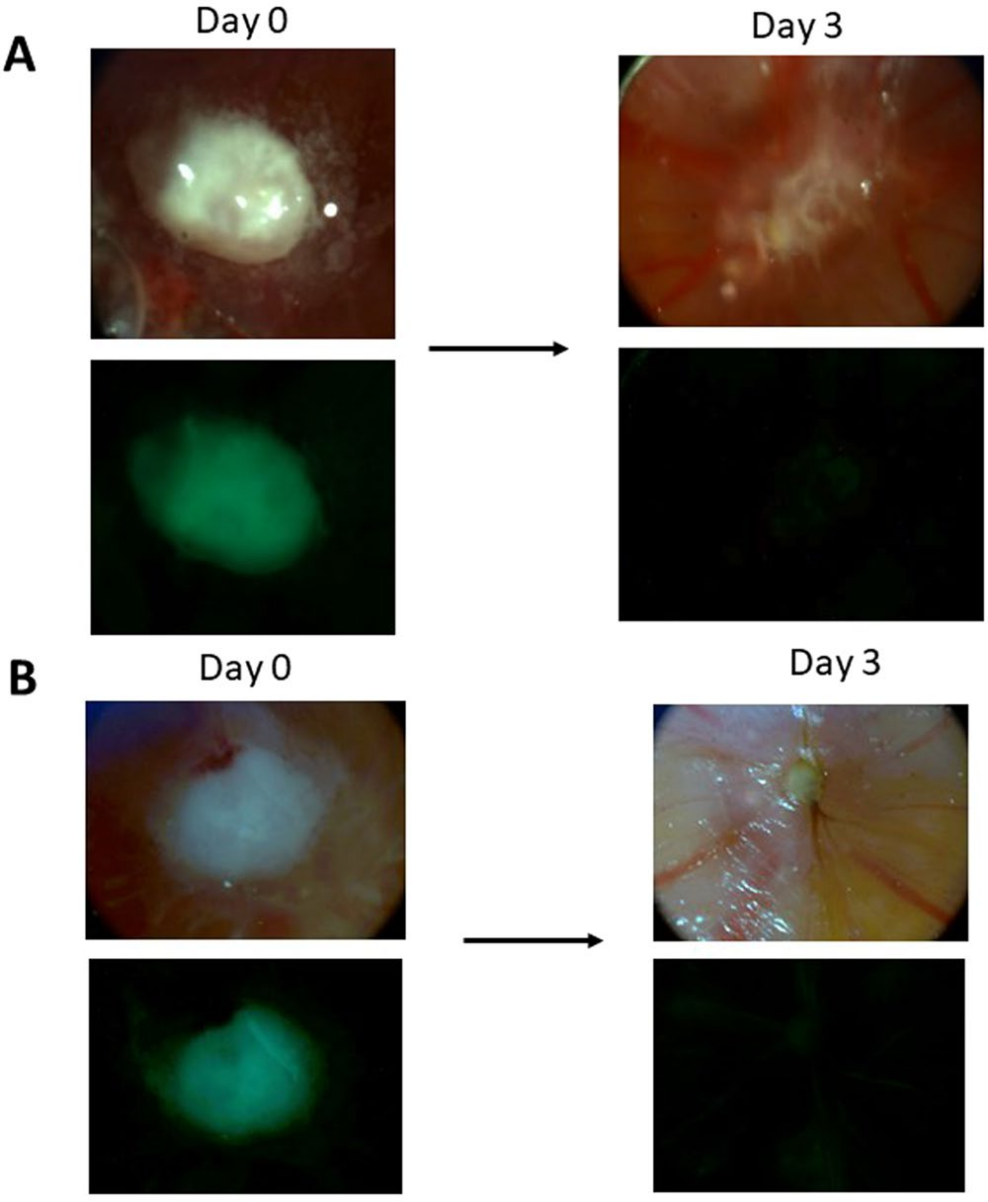
Intravenous injection of biodegradable PMO loaded with doxorubicin results in the elimination of Ov8GFP tumor. PMO-1 (**A**) or PMO-2 (**B**) loaded with doxorubicin was injected intravenously and the effect on tumor was examined by bright field and green fluorescence.

When the tumor was established on the CAM membrane, the biodegradable PMOs loaded with doxorubicin were administered intravenously into the chicken eggs (using a syringe as shown in Fig. 1) to examine effects of delivering doxorubicin. PMO nanoparticles were loaded with doxorubicin as described in Methods. Basically, we mixed doxorubicin solution with PMO nanoparticles, but to enhance the loading we used pH 5.5 condition as described by Croissant *et al*.^37^. Varying amounts (100 to 500 μg) of NPs were injected intravenously to chicken eggs with OVCAR8 tumor and the tumor was examined three days after the administration by the bright field as well as by green fluorescence using fluorescent stereomicroscope. Figure 4 shows the results of injecting PMO-1 (Fig. 4A) or PMO-2 (Fig. 4B) containing doxorubicin (the amount of nanoparticles was adjusted to deliver 200 μg of doxorubicin) and examined 3 days after injection. As can be seen, dramatic changes in the appearance of the tumor were observed indicative of tumor elimination. Green fluorescence confirmed that the tumor containing OVCAR8 cells was eliminated. Tumor shrinkage was observed even one day after the administration. The tumor regression was expected, as doxorubicin damages DNA and induces apoptosis.

### Lack of toxic effect when doxorubicin was loaded onto nanoparticles

**Figure 5:**
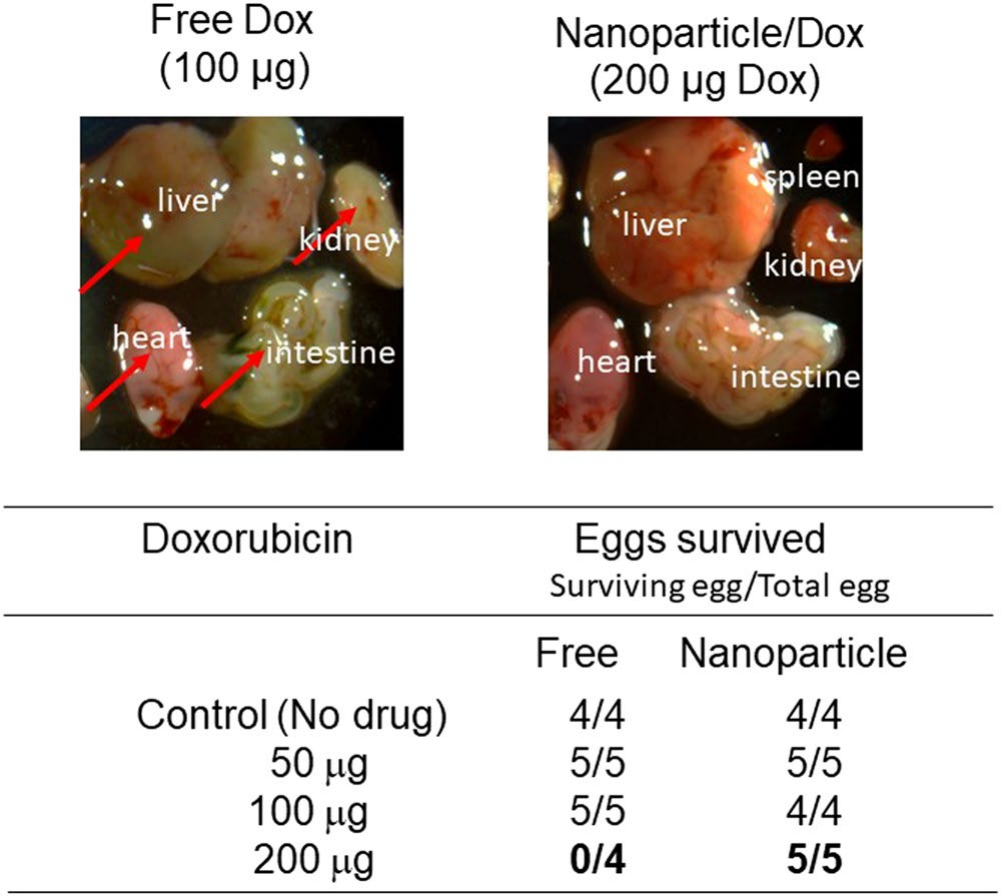
Lack of toxic effect by loading doxorubicin onto nanoparticle. PMO-1 loaded with doxorubicin or free doxorubicin was injected intravenously into chicken egg. Three days after the injection, internal organs were examined (Right panel: NP/Dox, Left panel: Free Dox). The bottom table shows survival of eggs after injection of free or nanoparticle formulated doxorubicin.

All eggs survived when injected with nanoparticles containing up to 200 μg of doxorubicin (Fig. 5). Internal organs (liver, heart, intestine, kidney and spleen) appeared normal after the treatment with nanoparticle carrying 200 μg of doxorubicin (Fig. 5 right picture). These results with nanoparticles were dramatically different from the results we obtained after administration of free doxorubicin. In the case of free doxorubicin, none of the eggs survived injection of 200 μg of doxorubicin. In addition, administration of 100 μg of doxorubicin caused significant damage to various organs in the embryo (Fig. 5 left picture). The color of liver, intestine and kidney was quite different. Kidney shape is altered and spleen shrank and could not be detected. These results suggest that free doxorubicin causes various adverse effect but that these side effect can be overcome by loading onto the nanoparticles.

### Doxorubicin is specifically delivered to the tumor after intravenous administration of doxorubicin loaded MSNs

**Figure 6:**
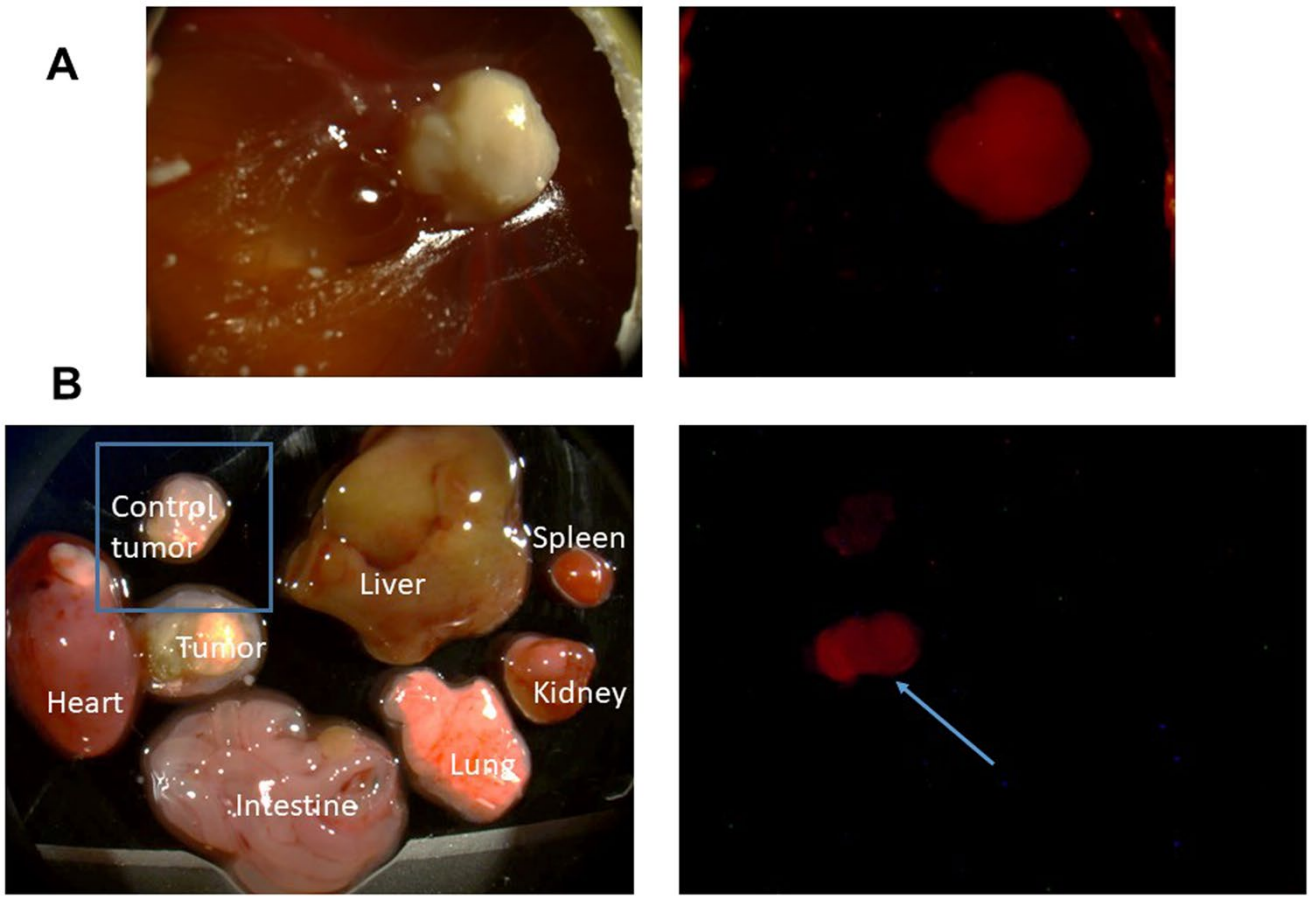
Tumor delivery of doxorubicin by nanoparticles. PMO-1 loaded with doxorubicin was intravenously injected and tumor delivery of doxorubicin was examined by following red fluorescence of doxorubicin (**A**). Internal organs were cut out and the presence of red fluorescence was examined (**B**).

The above results show that the nanoparticle formulation enables a higher amount of doxorubicin to be administered safely. To investigate tumor delivery of doxorubicin, we examined red fluorescence of doxorubicin in the tumor one and two days after injection of doxorubicin loaded PMO nanoparticles. As can be seen in Fig. 6A, the red fluorescence was observed in the Ov8GFP tumor. To examine whether the red fluorescence is observed predominantly in the tumor, various organs were removed, processed and examined by fluorescence microscopy. As can be seen in Fig. 6B, red fluorescence was observed predominantly in the tumor whereas little red fluorescence was observed in various organs from the embryo. Control tumor represents tumor sample obtained from eggs that did not receive nanoparticle injection. Thus, doxorubicin can be delivered preferentially in the tumor. In contrast, when free doxorubicin was administered, red fluorescence of doxorubicin was observed in all the organs examined (Supplementary Figure 1).

### Biodegradable PMO accumulates in the tumor

**Figure 7:**
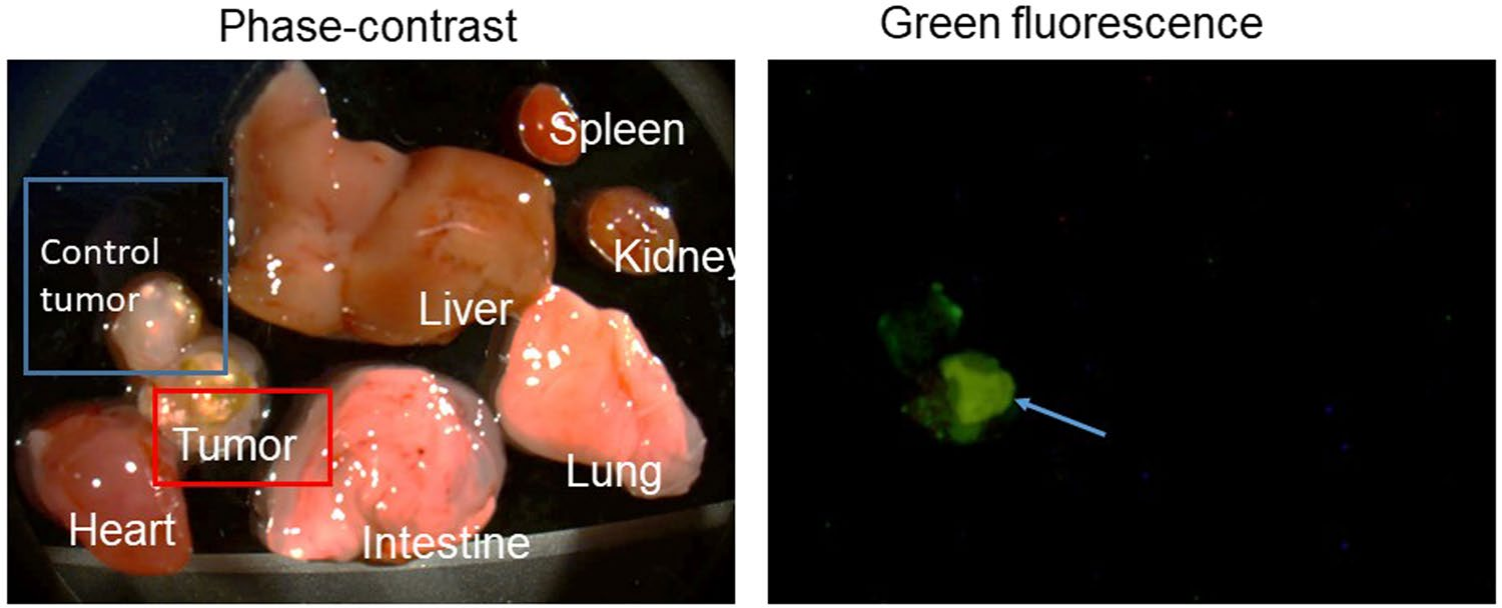
Tumor accumulation of PMO nanoparticles. FITC labeled PMO-2 were intravenously injected into eggs with A549 tumor. Three days later, tumor as well as various internal organs were isolated and examined.

To investigate whether the nanoparticles are localized to the tumor, we synthesized PMO nanoparticles labeled with FITC as described in Methods. These enable characterization of nanoparticle biodistribution by following green fluorescence. As for the chicken egg tumor, we used A549 lung cancer cells, as they do not express GFP and thus will not interfere with our analysis of nanoparticles. FITC labeled nanoparticles (0.5 mg) were injected and the distribution of green fluorescence was examined 1–3 days after the injection. Tumor and various organs from embryo were cut out. As can be seen in Fig. 7, green fluorescence was detected predominantly in the tumor three days after the injection. Green fluorescence was not detected in other organs such as liver. Control tumor represents tumor sample obtained from eggs that did not receive nanoparticle injection. Similar results were obtained when the analysis was carried out one day after the injection. Thus, biodegradable PMO nanoparticles preferentially accumulate in the tumor. On the other hand, biodegradable PMO surface modified to possess positive charge accumulated more in liver and kidney (see Discussion).

### Patient derived tumor model

**Figure 8:**
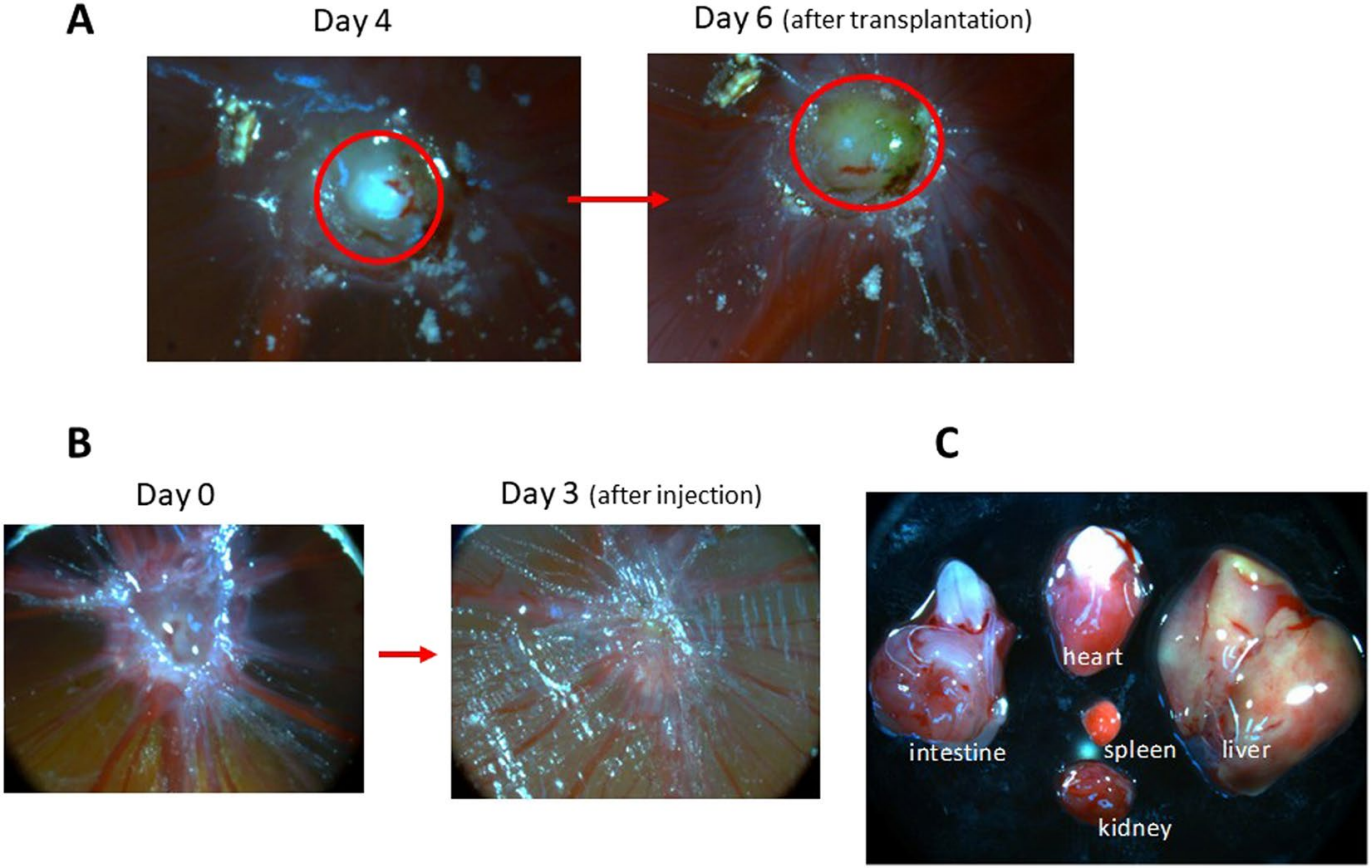
Patient tumor sample transplanted on the CAM membrane. (**A**) Tumor formed by transplanting minced sample of ovarian cancer patient tumor. (**B**) Tumor is eliminated after intravenous injection of PMO-1 containing doxorubicin. (**C**) Chick embryo major organs look normal three days after injection.

One of the attractive features of the chicken egg tumor model is that it is possible to use tumor tissue from ovarian cancer patients to transplant onto the CAM membrane. This enables evaluation of nanoparticle-based therapy using patient tumor. To explore this possibility, we used freshly obtained tumor from ovarian cancer patients. The sample was kept frozen, thawed and cut into small pieces of approximately 1 mm. These tumor pieces were placed on top of the CAM membrane and the eggs were incubated. As shown in Fig. 8A, we could clearly observe tumor formed by four days after transplantation and the tumor size grew by six days after transplantation.

Pathological analysis of patient samples (Fig. 2C) showed that they were high-grade serous carcinoma. Moreover, malignant cells were present, consistent with adenocarcinoma (CK7+/CK20-/CDX-2-/CA-125+). Histological analysis revealed P53 complete loss in invasive carcinoma and intraepithelial carcinoma; and strongly (contiguous) positive in other areas. Ki67 was elevated in invasive carcinoma and intraepithelial carcinoma (10–20%); low in other areas. Lastly, the reactive mesothelial cells were also seen within a background of blood and chronic inflammation.

We then injected PMO-1 nanoparticles loaded with doxorubicin intravenously (0.5–1 mg NP/egg) and followed effect on the tumor. As can be seen in Fig. 8B, the tumor was eliminated by three days after injection. With control experiments with no nanoparticle injection, tumor continued to grow. Examination of organs from the embryo after the nanoparticle injection showed that the organs are normal and are devoid of adverse effect by the treatment. Thus, the patient tumor model can be established and can be used for testing anticancer treatments.

## Discussion

In this paper, we have shown that a recently developed type of mesoporous silica nanoparticles called biodegradable PMO can exhibit excellent capability to delivery anticancer drugs in the chicken egg ovarian cancer model. One type of nanoparticles we used contain disulfide bonds^37^, while the other type of nanoparticle contains tetrasulfide bridges within the nanoparticle framework. These represent a new type of mesoporous silica nanoparticles that possess ideal property for further development, as these particles degrade upon exposure to reducing conditions that are encountered inside the cell. Both these nanoparticles could be loaded with doxorubicin and the drug could be delivered specifically to the tumor, resulting in the elimination of tumor. Gemcitabine, an FDA-approved chemotherapeutic drug used in the treatment of various cancers including ovarian cancer, could be loaded and delivered using PMO nanoparticles^42^. These results significantly advance the work on PMO by providing evidence for excellent property in animal settings.

Comparison of nanoparticle formulated doxorubicin with free doxorubicin demonstrates the advantages of nanoparticle formulation. When doxorubicin is loaded onto nanoparticles and injected into the bloodstream and targeted to the tumor, we did not observe any major adverse effect on chicken embryo. These results contrast with the administration of free drug where significant damage to various organs were observed. Nanoparticle formulation enables higher amount of doxorubicin to be used. We further demonstrated that doxorubicin is preferentially delivered to the tumor when the nanoparticle formulation was used, as shown by the preferential appearance of red fluorescence of doxorubicin in the tumor. This is in contrast with the distribution of red fluorescence when free doxorubicin was injected. In this case, we observed red fluorescence in all the organs examined. These results point to the tumor specific delivery of doxorubicin by the PMO nanoparticles.

The remarkable ability of our nanoparticles to deliver doxorubicin preferentially to the tumor reflects the ability of these nanoparticles to accumulate in the tumor. This was demonstrated by examining biodistribution of fluorescent nanoparticles after injection into chicken eggs. Delivery to various organs of the embryo was negligible. We are currently investigating what nanoparticle property contributes to this biological behavior. One possible contributing factor, we believe, is the negative surface charge of our nanoparticles, as changing the surface property to positive charge resulted in non-specific biodistribution of nanoparticles. As shown in Supplementary Figure 2, we surface coated PMO-2 nanoparticles with polyethylene imine (PEI) to provide positive surface charge. Injection of these nanoparticles to the chicken egg resulted in the presence of fluorescence in organs other than the tumor. In particular, strong fluorescence was observed in the liver and kidney. Presumably, negatively charged nanoparticles can have prolonged circulation and this enables tumor accumulation by taking advantage of the leaky vasculature (EPR, enhanced permeability and retention effect^43^). In fact, the tumor formed on the CAM membrane is quite vascularized, as we observe multiple blood vessels emanating from the tumor in this system. Prolonged circulation of our nanoparticles in the bloodstream presumably enables accumulation of nanoparticles in the tumor through leaky vasculature close to the tumor. Poor accumulation of various nanoparticles has been reported^44^. Comparison of our PMO nanoparticles with other types of nanoparticles need to be carried out, but this requires careful preparation of nanoparticles.

Our study demonstrates that the chicken egg tumor model provides a convenient and powerful system to evaluate the ability of nanoparticles to deliver anticancer drugs. Among various tumor models such as tumor organoid, 3D culture and mouse xenografts, this model stands out as an attractive system, as tumor formation is rapid presumably due to the incomplete establishment of the immune system; the immune system of the developing chick does not begin to function until about 2 weeks into its development^6,15,16^. We have shown that the tumor formed contains extracellular matrix and stromal cells and closely resemble the tumor in the patient.

Another important result of our work is that tumor samples from ovarian cancer patients can be used to establish tumor models in the chicken egg (Fig. 8). In this experiment, patient tumor is minced to small portions and transplanted onto the CAM membrane. Results demonstrate that tumor was formed in four days after transplantation and the patient-derived tumor grew in size in six days. Administration of free doxorubicin or nanoparticle-loaded doxorubicin eliminates the tumor burden. Thus, this patient derived chicken egg system (PDC tumor) provides a convenient assay to analyze patient tumor treatment options.

Precision medicine or personalized medicine has been promoted to provide tailor-made treatment to cancer patients. Therefore, it is necessary to use a patient-derived tumor model system to characterize possible treatment options. Currently, tumor organoids^13,14^ or patient-derived xenograft mouse systems (PDX model)^45^ are used. Based on our success to make patient derived tumor in the CAM model, we would like to suggest that the CAM model be added as an alternative model. This model can be called “patient derived chicken egg model (PDC model) and can be used to screen anticancer drugs to derive optimum drug for each individual cancer patient in a cost-effective manner. Thus, the PDC model could contribute to the advance of personalized medicine practice in the future.

## Methods

### Cell lines and culture media

OVCAR-8 cells were obtained from ATCC, and engineered to stably express a Green fluorescent protein (GFP)-firefly luciferase fusion protein, using the CMV-p:EGFP-ffluc pHIV7 vector (a gift from Christine Brown at City of Hope) to make the Ov8GFP line. Ov8GFP cells were maintained at 37 °C and 5% CO_2_ in a tissue culture incubator. Medium for the OVCAR-8 cells is RPMI 1640 medium supplemented with 10% fetal bovine serum and 1% penicillin/streptomycin. Cells were passaged using 0.25% trypsin every 2 days. In addition, a lung cancer cell line A549 cell line was obtained from ATCC. The cells were grown in DMEM supplemented with 10% FBS and 1% penicillin/streptomycin.

### Chicken egg tumor model

Freshly fertilized chicken eggs were purchased from McIntyre Farms (Lakeside, CA). The HovaBator Genesis 1588 Egg Incubator Combo Kit was purchased from Incubator Warehouse. 010 PTFE White Teflon O-Rings were purchased from The O-Ring Store. 3 M Tegaderm™ Transparent Dressing was purchased from Moore Medical and the Dremel 7700-1/15 MultiPro 7.2-Volt Cordless Rotary Tool Kit was used to make holes in the eggshell and 33 G acu·needles were purchased from Acuderm inc. Freshly fertilized chicken eggs were incubated at 100°F and with 60% humidity. On day 10 of embryo development, the chorioallantoic membrane (CAM) was dropped to make a window on the eggshell. A Teflon ring was placed on the CAM membrane which was then gently abraded with a stirring rod. 2 × 10^6^ OVCAR-8-GFP ovarian cancer cells were grafted into the ring, and then the window was sealed with Tegaderm film. Tumor formation was observed by a Leica MZ16F fluorescence stereomicroscope. All chicken egg experiments were approved by the UCLA Institutional Biosafety Committee, Office of Research Administration and were performed in compliance with the committee guideline. In ovo experiments do not require any special additional allowance as long as the embryos are sacrificed before hatching as is done in this study.

### Hematoxylin and eosin (H&E), trichrome and anti-Vimentin staining

For H&E staining, samples were processed in the Translational Pathology Core Laboratory (TPCL) in the UCLA Department of Pathology and Laboratory Medicine and a UCLA Jonsson Comprehensive Cancer Center Shared Facility. For trichrome staining, tumor thin sections were stained with the trichromic solution according to the protocol described by Andrea Monte-Alto-Costa and Luis Cristovao Porto^46^. Anti-Vimentin staining was carried out with polyclonal anti-Vimentin (Vim3B4) Mouse IgG2a (purchased from BioLegend) and goat Anti-Mouse IgG2a heavy chain (HRP) (Abcam).

### Preparation of biodegradable PMO nanomaterial

PMO-1 that contains disulfide bonds within the framework of nanoparticle was synthesized according to the procedure described by Croissant *et al*.^37^. Briefly, two different precursors, bis(triethoxysilylpropyl)disulfide and bis(triethoxysilyl)ethylene, were mixed (50%-50%) in the presence of NaOH and cetyltrimethylammonium bromide (CTAB). CTAB was removed after the synthesis. PMO-2 that contains tetrasulfide bonds was synthesized as follows. A mixture of CTAB (250 mg, 6.86 × 10^−1^ mmol), distilled water (120 ml), and sodium hydroxide (NaOH, 875 µl (2 M)) was stirred at 80 °C for 50 min at 1000 rpm in a 250 ml three neck round bottom flask. Then, the stirring speed was enhanced to 1400 rpm and 1,2-bis(triethoxysilyl)ethane (300 µl, 8.1 × 10^−1^ mmol) was added, and followed by bis(3-triethoxysilylpropyl)tetrasulfide (100 µl, 2.0 × 10^−1^ mmol). The condensation process was conducted for 2 h. Then, the solution was cooled to room temperature while stirring; fractions were gathered in propylene tubes and collected by centrifugation for 15 min at 14,000 rpm. The prepared PMO sample was sonicated twice with an ethanolic solution of ammonium nitrate (6 g·l^−1^) and washed three times with ethanol, water, and ethanol. Each washing step involved a sonication of 20 min at 40 °C and nanoparticles were collected by centrifugation. The as-prepared CTAB-free PMO nanomaterial was finally dried under vacuum for a few hours. The PMO nanoparticles were further surface modified as follows. PMO nanomaterial (127.2 mg) was redispersed in distilled water (29.6 ml). Then, 3-(trihydroxysilyl)propyl methylphosphonate (59.2 μl, 15.56 × 10^−2^ mmol) was added to aqueous dispersion of PMO NPs and stirred for 3 h at 60 °C. After this time, dispersion was cooled to room temperature while stirring, and the nanomaterial was collected and washed as described above. FITC labeled PMO nanoparticles were prepared by the co-condensation with FITC-APTES prepared by chemically linking fluorescent isothiocyanate (FITC) dye with aminopropyltriethoxysilane (APTES).

### Loading of doxorubicin

30 mg of nanoparticles were suspended in 3 ml H_2_O with 9.4 mg of doxorubicin (DOX) at pH 5.5. The suspension was sonicated for 5 min at 45 °C then stirred at 370 rpm for 24 h at RT. The pH was then adjusted to 7 and the suspension stirred for 30 min. The suspension was centrifuged and the nanoparticles were washed with H_2_O. As reported earlier^37^, biodegradable PMO has a high loading capacity for doxorubicin when pH 5.5 condition was used. The amount of doxorubicin ranged between 20 and 50% to total weight. In one experiment using PMO-1, we observed that doxorubicin constituted 47% of the total weight of the loaded nanoparticle.

### Nanoparticle and nanoparticle/doxorubicin injection into CAM model

Three days post tumor cell inoculation, CAM blood vessel was identified using flashlight, a small window was made on the shell right above the labelled blood vessel. The window was cut out using Dremel tool to keep the inner eggshell intact. One drop of immersion oil was put onto the inner eggshell to facilitate observation of the blood vessel. 0.1 ml of nanoparticle or nanoparticle/doxorubicin solution was injected into CAM blood vessel using 33 G needle.

### Ovarian cancer patient tumor sample

Fresh tumor sample from ovarian cancer patients were frozen in Recovery^TM^-Cell culture freezing medium from Gibco (Life Technologies). The sample was thawed and minced with scalpel and scissors into suspension which was then grafted onto CAM membrane as describing in chicken egg tumor model method. All research was performed in accordance with relevant guidelines/regulations as approved by the City of Hope committee under IRB#15027. Informed consent was obtained from all subjects.

Materials and Data obtained in this study are deposited to the publisher site and are available upon request.

## Electronic supplementary material

Supplementary Info
