# Supplementary material for: Chick chorioallantoic membrane assay as an in vivo model to study the effect of nanoparticle-based anticancer drugs in ovarian cancer
Source: Sci Rep. 2018 Jun 4;8:8524. doi: 10.1038/s41598-018-25573-8 (PMC5986798; doi:10.1038/s41598-018-25573-8)
Supplement: Supplementary file 1 — Supplementary Info [file 41598_2018_25573_MOESM1_ESM.pdf]

Supplementary Information

Chick chorioallantoic membrane assay as an *in vivo* model to study the effect of nanoparticle-based anticancer drugs in ovarian cancer

Binh Thanh Vu[1], Sophia Allaf Shahin[2], Jonas Croissant[3], Yevhen Fatieviev[3], Kotaro Matsumoto[4], Tan Le Hoang Doan[4], Tammy Yik[1], Shirleen Simargi[2], Altagracia Conteras[2], Laura Ratliff[2], Chiara Mauriello Jimenez[5], Laurence Raehm[5], Niveen Khashab[3], Jean-Olivier Durand[5], Carlotta Glackin[2] and Fuyuhiko Tamanoi[1,4]

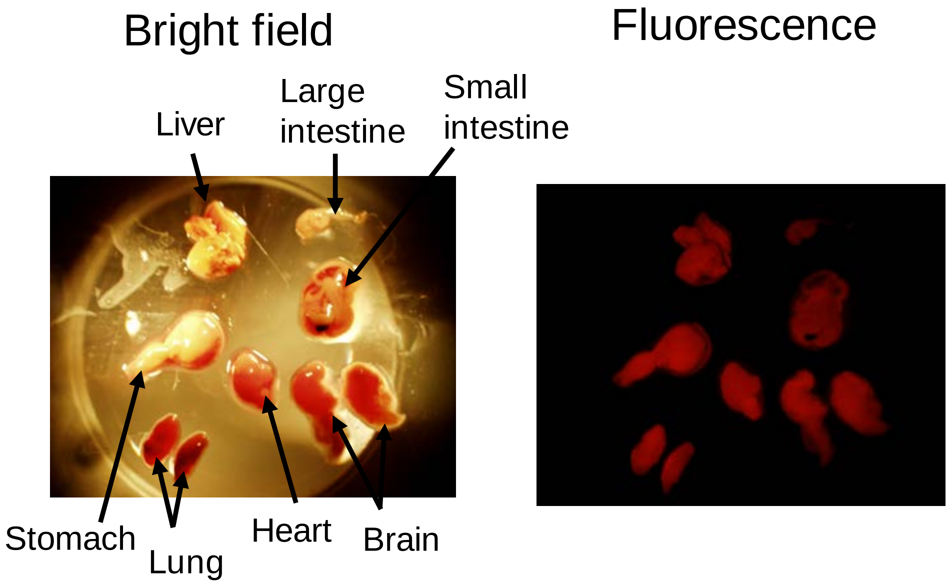

Supplementary Figure 1

Supplementary Figure 1: Non-specific delivery of free doxorubicin. Ov8GFP cells were used to establish tumor on the CAM membrane. Doxorubicin (50 μg) was injected intravenously and red fluorescence of internal organs was examined three days after the injection. Bright field (Left) and fluorescence field (right) are shown.

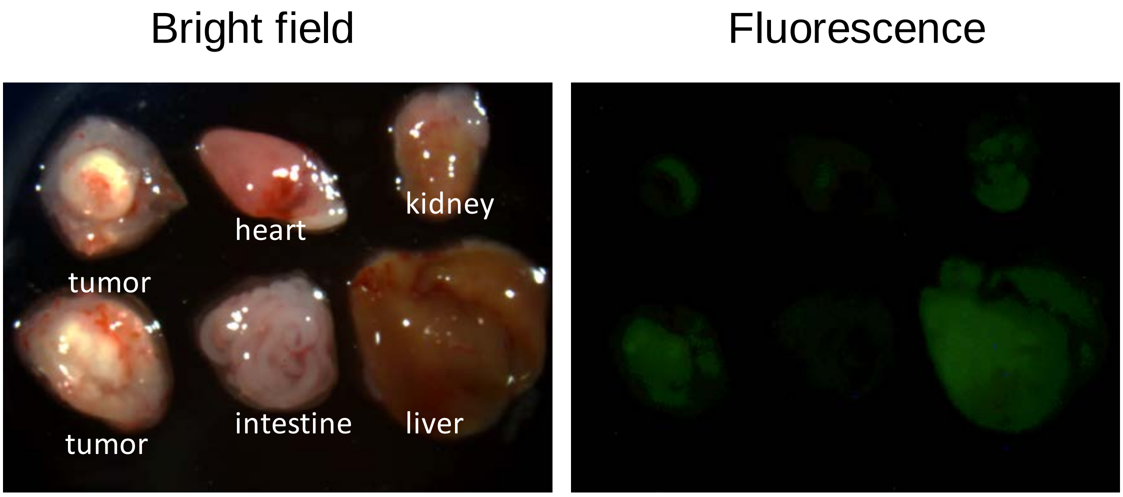

Supplementary Figure 2

Supplementary Figure 2: Non-specific biodistribution of PEI-coated PMO-2 nanoparticles. FITC labeled PMO-2 nanoparticles were surface coated with polyethyleneimine (PEI) according to the procedure described by Finlay et al (Nanomedicine 11, 1657, 2015)[29]. In brief, low molecular weight (1.8 kD branched polymer) polyethyleneimine (PEI) was electrostatistically attached to the PMO-2 nanoparticle surface to provide positive charge. 0.5 mg of PEI-PMO-2 nanoparticles were injected intravenously into chicken egg with A549 tumor. Three days after the injection, various organs as well as tumor were cut out and biodistribution of PEI-PMO-2 was examined by green fluorescence of the nanoparticles.
